# Modulation of Pyruvate Export and Extracellular Pyruvate Concentration in Primary Astrocyte Cultures

**DOI:** 10.1007/s11064-024-04120-0

**Published:** 2024-02-20

**Authors:** Nadine Denker, Ralf Dringen

**Affiliations:** Centre for Biomolecular Interactions Bremen, Faculty 2 (Biology/Chemistry) and Centre for Environmental Research and Sustainable Technologies, University of Bremen, P.O. Box 330440, 28334 Bremen, Germany

**Keywords:** Astrocytes, Metabolism, Mitochondria, MCT1, Pyruvate, Transport

## Abstract

Astrocyte-derived pyruvate is considered to have neuroprotective functions. In order to investigate the processes that are involved in astrocytic pyruvate release, we used primary rat astrocyte cultures as model system. Depending on the incubation conditions and medium composition, astrocyte cultures established extracellular steady state pyruvate concentrations in the range between 150 µM and 300 µM. During incubations for up to 2 weeks in DMEM culture medium, the extracellular pyruvate concentration remained almost constant for days, while the extracellular lactate concentration increased continuously during the incubation into the millimolar concentration range as long as glucose was present. In an amino acid-free incubation buffer, glucose-fed astrocytes released pyruvate with an initial rate of around 60 nmol/(h × mg) and after around 5 h an almost constant extracellular pyruvate concentration was established that was maintained for several hours. Extracellular pyruvate accumulation was also observed, if glucose had been replaced by mannose, fructose, lactate or alanine. Glucose-fed astrocyte cultures established similar extracellular steady state concentrations of pyruvate by releasing pyruvate into pyruvate-free media or by consuming excess of extracellular pyruvate. Inhibition of the monocarboxylate transporter MCT1 by AR-C155858 lowered extracellular pyruvate accumulation, while inhibition of mitochondrial pyruvate uptake by UK5099 increased the extracellular pyruvate concentration. Finally, the presence of the uncoupler BAM15 or of the respiratory chain inhibitor antimycin A almost completely abolished extracellular pyruvate accumulation. The data presented demonstrate that cultured astrocytes establish a transient extracellular steady state concentration of pyruvate which is strongly affected by modulation of the mitochondrial pyruvate metabolism.

**Supplementary Information:**

The online version contains supplementary material available at 10.1007/s11064-024-04120-0.

## Introduction

Astrocytes play an important role in brain energy metabolism [1–4], but have also crucial functions in brain development [5], (ion) homeostasis [6–9], the regulation and modulation of neuronal signals [10, 11], memory formation [12] and the protection against toxins and oxidative stress [13–15]. Although astrocytes are considered as a rather glycolytic cell type [16], also the oxidative metabolism plays an important role for astrocytic energy regeneration [17–19]. In this context, the α-ketoacid pyruvate is of high interest as it links cytosolic glycolysis and mitochondrial metabolism [17, 20, 21]. Pyruvate, the end product of glycolysis, can be taken up into mitochondria via the proton-coupled mitochondrial pyruvate carrier (MPC) [22–24], and subsequently be decarboxylated to acetyl-CoA via the pyruvate dehydrogenase complex [17, 25], or carboxylated to oxaloacetate by pyruvate carboxylase [26–28]. As the rate of pyruvate decarboxylation in mitochondria is slow in astrocytes [29], the cytosolic reduction of pyruvate by lactate dehydrogenase (LDH) is a favoured reaction at least in cultured astrocytes in order to regenerate NAD^+^ for further glycolytic glucose degradation [30, 31]. Extracellular pyruvate can be efficiently taken up and metabolized by cultured astrocytes [18, 32, 33]. However, such cultures have also been reported to release pyruvate [34–36]. Pyruvate transport over the astrocytic plasma membrane is mainly mediated by proton-coupled monocarboxylate transporters (MCTs) [37–40].

Extracellular pyruvate has been shown to be neuroprotective in models of glutamate-toxicity [41, 42], oxidative stress [36, 43] and ischemia [44] and several potential mechanisms have been discussed that may contribute to this neuroprotective function [45]. For brain, extracellular pyruvate concentrations of around 160 µM have been reported [46, 47]. Although astrocytic pyruvate export is likely to contribute to this extracellular pyruvate pool, little is known so far on the metabolic processes and pathways that modulate pyruvate release from astrocytes.

We have previously reported that cultured rat astrocytes efficiently consume extracellular pyruvate in the absence of glucose [32, 33] in a process that depends on MCT1 and MPC and is strongly modulated by mitochondrial activity [33]. For our current study, we have investigated the pyruvate release from glucose-fed cultured astrocytes and have tested for the involvement of potential transporters and/or metabolic pathways that modulate pyruvate export and the extracellular pyruvate concentration. Here we report that cultured astrocytes establish transient extracellular steady state concentrations of pyruvate in a concentration range between around 150–300 µM, which are independent from the initial extracellular pyruvate concentration applied. The extracellular pyruvate concentration was increased by inhibition of mitochondrial pyruvate uptake, but lowered after inhibition of MCT1 or by application of the mitochondrial uncoupler BAM15 or of the respiratory chain inhibitor antimycin A. These data demonstrate that pyruvate release and the extracellular concentration of pyruvate are strongly affected by a modulation of mitochondrial pyruvate metabolism.

## Materials and Methods

Penicillin G / streptomycin sulfate solution and powder to prepare Dulbecco’s modified Eagles medium (DMEM with 25 mM glucose; catalog number: 52100-021) were obtained from Thermo Fisher Scientific (Schwerte, Germany; RRID:SCR_008452). Glucose-free DMEM powder (catalog number: D5030), fetal calf serum (FCS), antimycin A, BAM15 and UK5099 were purchased from Sigma-Aldrich (Darmstadt, Germany; RRID:SCR_008988). AR-C155858 was purchased at Tocris Bioscience (Bristol, UK; RRID:SCR_003689). All enzymes used for the assays to determine pyruvate, lactate and glucose were purchased from Roche Diagnostics (Mannheim, Germany; RRID:SCR_001326). Other chemicals of the highest purity available were obtained from Merck (Darmstadt, Germany; RRID:SCR_001287), Sigma-Aldrich (Steinheim, Germany; RRID:SCR_008988), AppliChem (Darmstadt, Germany; RRID:SCR_005814) or Carl Roth (Karlsruhe, Germany; RRID:SCR_005711). Sterile cell culture materials and unsterile 96-well plates were from Sarstedt (Nümbrecht, Germany).

### Astrocyte Cultures

Astrocyte-rich primary cultures were prepared as previously described in detail from the total brains of newborn Wistar rats [48]. From the harvested cell suspension, 300,000 cells were seeded per well of 24-well dishes in 1 mL culture medium (90% DMEM containing 25 mM glucose, 44.6 mM sodium bicarbonate, 1 mM pyruvate, 20 U/mL penicillin G, 20 µg/mL streptomycin sulfate, supplemented with 10% FCS). The cultures were maintained in a humidified atmosphere with 10% CO_2_ in a Sanyo CO_2_ incubator (Osaka, Japan). The culture medium was renewed every seventh day and one day prior to an experiment. If not stated otherwise, confluent astrocyte cultures of an age between 19 and 27 days were used for experiments. It should be noted here, that the specific pyruvate export from cultured astrocytes was found to be lowered to some extend with increasing culture age, while the glucose consumption and lactate release were not affected by the cultured age (Fig. S1). Astrocyte-rich primary cultures are strongly enriched in astrocytes and contain only low amounts of contaminating microglial cells and oligodendrocytes [48, 49].

### Experimental Incubation of the Cells

**Fig. 1:**
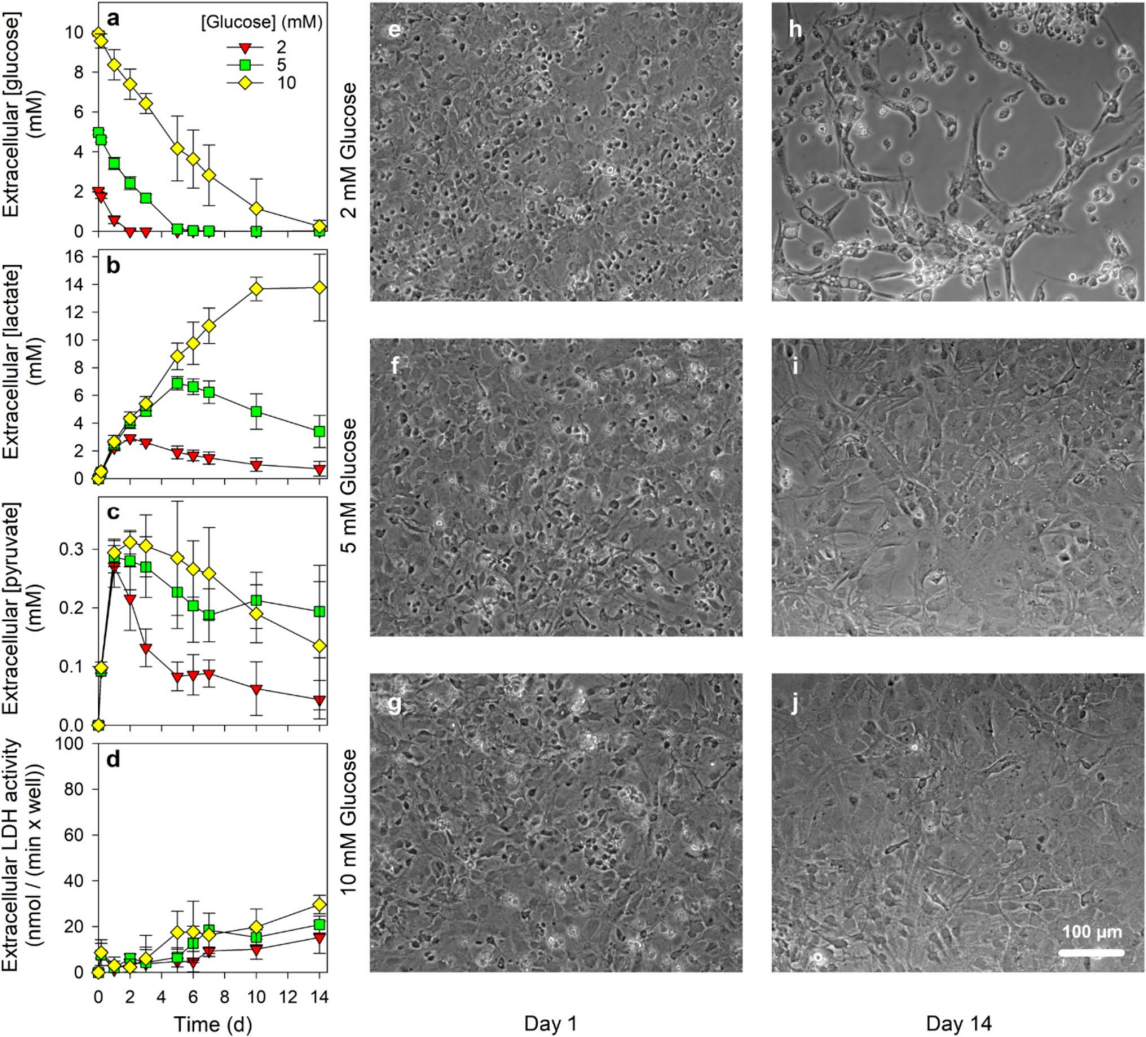
Glucose consumption and extracellular accumulation of pyruvate and lactate in primary astrocyte cultures. Astrocyte cultures were incubated for up to 14 days in serum-free DMEM that contained the initial glucose concentrations indicated in panel a. The extracellular concentrations of glucose (**a**), lactate (**b**) and pyruvate (**c**) as well as the extracellular LDH activity (**d**) as an indicator of a potential loss in cell viability were measured for the indicated time points. The initial cellular LDH activity of the cultures at the onset of the incubation (100%) was 106 ± 19 nmol/(min × well) and the initial protein content was 121 ± 55 µg/well. The data presented are means ± SD of data obtained in three individually performed experiments on independently prepared cultures. Panels **e**–**g** show representative phase-contrast pictures of the cultures after incubation for 1 day, while panels h to j show pictures of the same cultures after 14 days of incubation

For long-time incubations of up to 14 d (Fig. 1), the culture medium was aspirated from the cultures (age of 13 or 14 d), the cells were washed twice with 1 mL pre-warmed (37 °C) glucose-free DMEM (containing 44.6 mM sodium bicarbonate, 20 U/mL penicillin G and 20 µg/mL streptomycin sulfate) and were subsequently incubated with 1 mL of glucose-free DMEM that had been supplemented with glucose in the indicated concentrations. After the given incubation periods the incubation medium was harvested for determination of the cell viability and the extracellular concentrations of glucose, lactate and pyruvate.

For short-time incubations of up to 12 h, the cells in the cultures (age between 19 and 27 d) were washed twice with 1 mL incubation buffer (IB; 145 mM NaCl, 20 mM HEPES, 5.4 mM KCl, 1.8 mM CaCl_2_, 1 mM MgCl_2_, 0.8 mM Na_2_HPO_4_, pH adjusted with NaOH to 7.4 at 37 °C) and subsequently incubated for the time periods indicated at 37 °C in the humidified atmosphere of a CO_2_-free incubator with 250 µL of IB that had been supplemented with glucose, other substrates, inhibitors of transporters and/or modulators of metabolic pathways, if not indicated otherwise. For all supplements that had been dissolved as concentrated stock solutions in DMSO, appropriate solvent controls were performed that confirmed that the final concentration of DMSO present during the incubation did not affect the parameters investigated (data not shown). After the given incubation periods the incubation medium was harvested for determination of potential LDH release (as indicator for cell toxicity) and of the extracellular concentrations of glucose, lactate and/or pyruvate.

### Determination of Cellular Protein and Cell Viability

For determination of the cellular protein content per well the cultures were washed twice with 1 mL ice-cold (4 °C) phosphate-buffered saline (PBS; 10 mM potassium phosphate buffer pH 7.4 containing 150 mM NaCl) and stored frozen until the protein determination was performed by the Lowry method [50] using bovine serum albumin as standard protein. To test for potential cell toxicity of a given treatment the extracellular activity of the cytosolic enzyme LDH was determined after the treatment for 10 µL media samples and compared with the initial cellular LDH activity of untreated cells, as previously described in detail [48].

### Determination of Extracellular Pyruvate, Glucose and Lactate

Pyruvate was determined by a photometric microtiter plate assay using the LDH- and NADH-dependent reduction to lactate by a modification [33] of the method described previously by Clarke and Payton [51]. The concentration of extracellular glucose was determined by a coupled enzymatic assay using hexokinase and glucose-6-phosphat dehydrogenase as previously described in detail [48]. Glucose and pyruvate consumptions were calculated as difference between the concentrations applied and the concentrations determined after a given incubation period. Extracellular lactate was quantified by a coupled enzymatic assay using LDH and glutamate-pyruvate transaminase in an alkaline glutamate buffer as previously described in detail [48].

### Determination of Cellular Lactate

Cellular lactate content was determined in neutralized perchlorate lysates of cultured astrocytes [33]. Briefly, the cells were washed twice with 1 mL ice-cold PBS on ice and lysed with 100 µL ice-cold 0.25 M HClO_4_ per well. Subsequently, the cell lysates from two wells were collected and pooled. The cell lysates were neutralized by addition of an appropriate amount of 2 M KOH to a pH of 7 and centrifuged for 5 min at 12,100×*g* to precipitate the KClO_4_ formed. Of the lysate supernatant, 190 µL were mixed with 10 µL of alkaline glutamate buffer (500 mM glutamate buffer pH 8.9, adjusted with NaOH) and 180 µL of the mixture was used to quantify lactate by a coupled enzymatic assay with LDH and glutamate-pyruvate transaminase [48].

### Presentation of Data and Statistical Analysis

Quantitative data are shown as means ± SD of values that have been obtained from three individual experiments performed on independently prepared astrocyte cultures. For this low number of individual experiments, statistical analysis was done under the assumption of normal distribution. Analysis for statistical significance of groups of data was performed by ANOVA followed by the Bonferroni post-hoc test using the software GraphPad InStat (GraphPad, Boston, USA; RRID:SCR_000306). The paired *t*-test was used to calculate the statistical significance between pairs of data. The level of significance of differences compared to the data obtained for the respective control condition or between pairs of data is indicated by the symbols given in the legends of the individual figures. A p-value above 0.05 was considered as not significant.

## Results

### Extracellular Pyruvate and Lactate Accumulation in Cultured Primary Astrocytes During a Long-Time Incubation for 14 d

To investigate the extracellular accumulation of pyruvate and lactate during a long-time incubation of cultured astrocytes to media that contained limited concentrations of glucose, the cells were exposed to an initial glucose concentration of 2 mM, 5 mM or 10 mM in DMEM incubation medium and the extracellular concentrations of glucose, lactate and pyruvate as well as the cell viability were monitored for an incubation period of up to 14 d. After exposure of cultured astrocytes to a given concentration of glucose, the cells efficiently consumed the available glucose from the medium (Fig. 1a). For media containing glucose in initial concentrations of 2 mM, 5 mM and 10 mM, the detectable glucose had been almost completely metabolized within 2 d, 5 d and 14 d (Fig. 1a), respectively. This cell-dependent metabolic glucose depletion was accompanied by a rapid increase in the extracellular concentration of lactate, which reached for media that contained initially 2 mM, 5 mM and 10 mM glucose maximal lactate value of around 3 mM (after 2 d), around 7 mM (after 5 d) and of around 13 mM (after 10 d), respectively, representing around 150% of the concentration of glucose initially applied (Fig. 1b). In contrast, extracellular pyruvate accumulated for all glucose concentrations applied within 24 h to an extracellular concentration of around 0.3 mM (Fig. 1c). For cultures that had been fed with 10 mM glucose, extracellular pyruvate concentrations above 0.2 mM were maintained for several days, while extracellular pyruvate concentrations of cultures that had been exposed to initial glucose concentrations of 2 mM or 5 mM declined earlier (Fig. 1c). For all conditions, pyruvate levels were lowered during incubations (Fig. 1c) already before the extracellular lactate concentrations started to decline (Fig. 1b).

The viability of the cells, as demonstrated by the absence of a substantial increase in extracellular LDH activity (Fig. 1d) and by inspection of the cell morphology (Fig. 1e–g), was not compromised during the initial phase of the incubation for all the conditions applied. However, damage in the confluent cell layer was observed after a 14 d-incubation of cultures that had been exposed to only 2 mM glucose (Fig. 1h), but not for cultures that had been incubated with media that contained initially 5 mM or 10 mM glucose (Fig. 1i, j). Nevertheless, for the latter treatments the majority of bright cells on top of the basal astrocyte cell layer, that had been visible in the cultures after 1 d of treatment (Fig. 1e–g), disappeared during the incubation for 14 d (Fig. 1i, j), suggesting that some cells in the culture may have not survived the respective treatments.

Application of pyruvate in an initial concentration of 0.5 mM to cultured astrocytes in DMEM containing initial glucose concentration of 2 mM, 5 mM or 10 mM resulted in almost identical results on glucose consumption, extracellular lactate accumulation and cell viability (Fig. S2a, c, d), compared to those recorded for the respective incubations without initial pyruvate application (Fig. 1a, b, d). In the glucose-containing media extracellular pyruvate concentrations between 0.3 and 0.4 mM were established within 2 d that were found further lowered during longer incubations (Fig. S2b). For longer incubations in glucose-containing media, almost identical extracellular pyruvate concentrations were found (Fig. S2b) as those determined for astrocyte cultures that had been exposed to the respective media that did not contain initial pyruvate (Fig. 1c).

### Glucose-Dependent Extracellular Pyruvate Accumulation in Short-Time Experiments

**Fig. 2:**
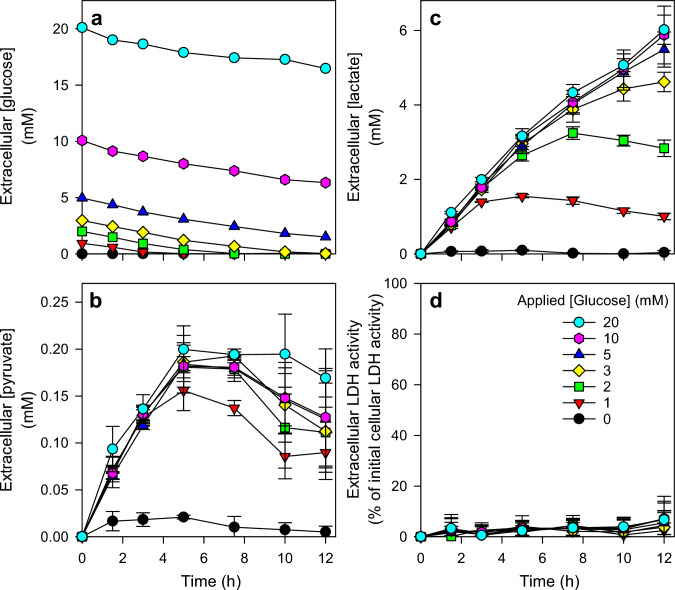
Glucose-dependency of pyruvate and lactate release from cultured astrocytes. Cultured primary astrocytes were incubated for up to 12 h in incubation buffer containing the initial glucose concentrations indicated in panel d. For the given time points the concentrations of extracellular glucose (**a**), pyruvate (**b**) and lactate (**c**) were measured. In addition, extracellular LDH activity (**d**), as an indicator of a potential loss in cell viability, was determined. The initial cellular LDH activity of the cultures was 159 ± 17 nmol/(min × well) and the initial protein content was 137 ± 11 µg/well. The data shown represents means ± SD of values derived from experiments performed on three independently prepared cultures

To investigate a potential glucose dependency of pyruvate release from astrocytes in a short-time setting, the cultures were incubated in 250 µL of HEPES-buffered incubation buffer (IB) containing different initial concentrations of glucose and the extracellular concentrations of glucose, lactate and pyruvate were monitored during incubations for up to 12 h. During these incubations, glucose was consumed (Fig. 2a) while pyruvate (Fig. 2b) and lactate (Fig. 2c) accumulated extracellularly. For glucose concentrations above 3 mM, the decline in extracellular glucose (Fig. 2a) as well as the extracellular accumulation of lactate (Fig. 2c) were almost proportional to the incubation time as long as glucose was present. For initial glucose concentrations of 3 mM and below, the applied glucose was found completely metabolized before the end of the 12 h incubation (Fig. 2a) which limited the extracellular lactate accumulation (Fig. 2c). In contrast, for all the initial concentrations of glucose applied, extracellular pyruvate accumulated with a similar initial specific accumulation rate during the first hours of around 60 nmol/(h × mg), reaching after 5 h maximal extracellular pyruvate concentrations of 150 to 200 µM (Fig. 2b) that were maintained for several hours before they were lowered during longer incubations. None of the conditions applied had any obvious toxic potential as indicated by the absence of any LDH release from the cells during the 12 h incubation (Fig. 2d).

### Similar Extracellular Concentrations of Pyruvate are Established by Pyruvate Export and/or Pyruvate Consumption

**Fig. 3:**
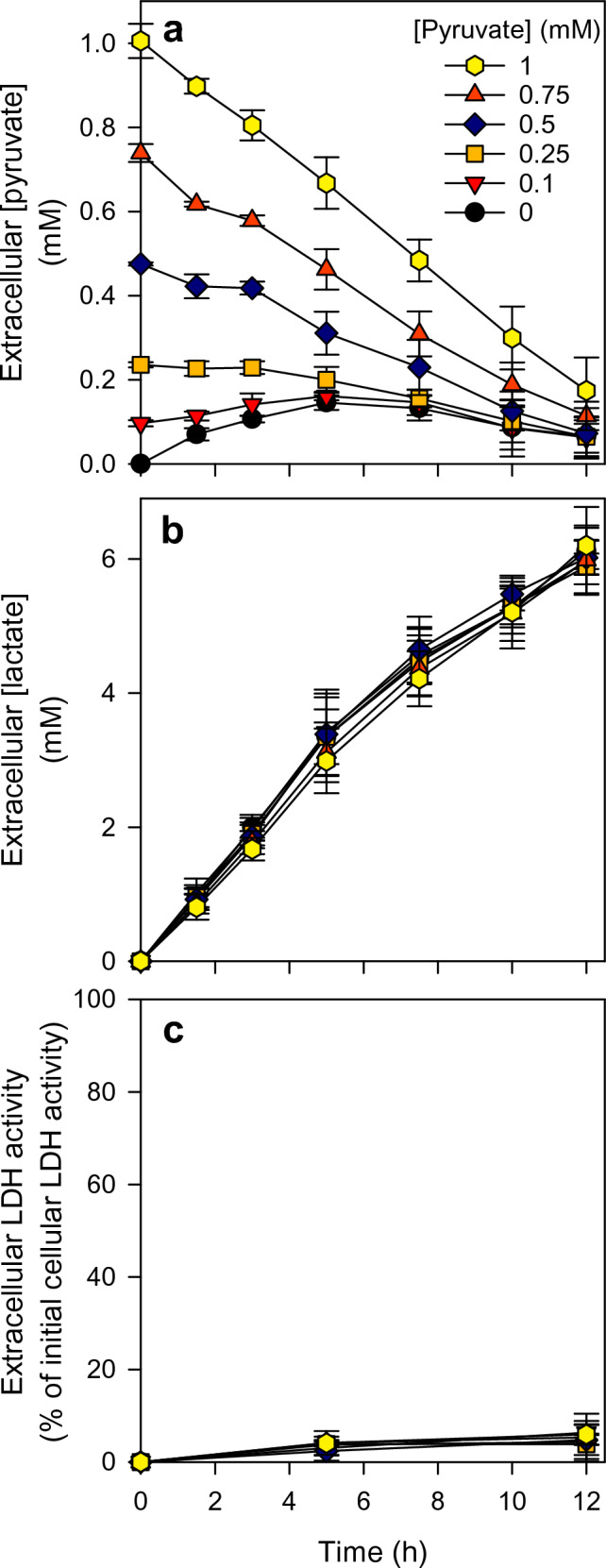
Extracellular pyruvate concentrations in glucose-fed astrocyte cultures after application of different initial pyruvate concentrations. The cultures were incubated with 20 mM glucose in the absence (0 mM) or the presence of pyruvate in the concentrations indicated (panel a) for up to 12 h. For the time points indicated extracellular concentrations of pyruvate (**a**) and lactate (**b**) as well as the extracellular LDH activity (**c**) as an indicator for potential cell toxicity were measured. The initial cellular LDH activity of the cultures was 150 ± 5 nmol/(min × well) and the initial protein content was 150 ± 8 µg/well. The data shown are means ± SD from three individual experiments performed on independently prepared cultures

Astrocytes have been reported to efficiently consume extracellularly applied pyruvate [33]. To test how the initial application of an excess of pyruvate may affect pyruvate release and/or the extracellular pyruvate concentration in glucose-fed astrocytes, the cells were exposed to glucose in the absence or the presence of pyruvate in different initial concentrations of up to 1 mM (Fig. 3). None of the conditions applied had any obvious toxic potential as indicated by the absence of any LDH release from the cells during the incubation (Fig. 3c). Astrocytes that were incubated without or with 0.1 mM pyruvate, exported pyruvate during the incubation and the extracellular pyruvate concentrations were found to increase to values of around 150 µM within 5 h of incubation (Fig. 3a). In contrast, if pyruvate had been applied to the cells in initial concentrations above 0.2 mM, the extracellular pyruvate concentrations declined during the incubation and reached within 12 h of incubation extracellular concentrations between 100 µM and 200 µM (Fig. 3a). In contrast, for all pyruvate concentrations applied, glucose-fed astrocytes released lactate during the incubation at a rate that was almost proportional to the incubation time (Fig. 3b), causing an extracellular lactate accumulation to a concentration of around 6 mM within 12 h (Fig. 3b).

### Test for a Potential Protection of Extracellular Pyruvate by Antioxidative Enzymes

Pyruvate has been reported to be efficiently oxidized to acetate by the presence of hydrogen peroxide [52]. For peripheral cell lines, the detectable extracellular pyruvate level has been reported to be lowered to some extent by such a reaction [53]. As the release of small amounts of hydrogen peroxide has been reported for an astroglial cell line [54] as well as for cultured primary and secondary astrocytes [55–57], we tested whether extracellular oxidation of pyruvate by cell-generated extracellular hydrogen peroxide may also lower the detectable extracellular pyruvate concentration in astrocyte cultures. The cells were incubated for 5 h with 5 mM glucose without or with 1 mM pyruvate in the absence or the presence of catalase and/or superoxide dismutase (SOD) to efficiently remove extracellular superoxide and hydrogen peroxide during the incubation, as previously shown for cultured astrocytes [58, 59]. However, the extracellular presence of the enzymes did not alter the extracellular pyruvate or lactate concentrations determined for cultures that had been incubated in the absence or the presence of 1 mM pyruvate (Table 1). None of the conditions applied had any obvious toxic potential as indicated by the absence of any LDH release from the cells during the incubation (Table 1). Thus, for the conditions used a potential chemical oxidation of released pyruvate by cell-derived hydrogen peroxide appears not to affect the cell-derived extracellular pyruvate levels.

**Table 1:** Test for peroxide-mediated pyruvate degradation in cultured astrocytes

		None	SOD	Catalase	Catalase + SOD
0 mM Pyruvate	[Pyruvate] (µM)	122 ± 8	126 ± 14	136 ± 10	125 ± 9
	[Lactate] (mM)	3.87 ± 0.07	3.69 ± 0.16	3.53 ± 0.19	3.59 ± 0.14
	Extracellular LDH (% of initial cellular LDH)	7 ± 3	3 ± 2	5 ± 4	4 ± 4
1 mM Pyruvate	[Pyruvate] (µM)	573 ± 25	613 ± 55	591 ± 28	586 ± 51
	[Pyruvate] consumed (µM)	347 ± 90	358 ± 74	404 ± 56	399 ± 109
	[Lactate] (mM)	3.48 ± 0.05	3.26 ± 0.26	3.22 ± 0.05	3.22 ± 0.03
	Extracellular LDH (% of initial cellular LDH)	11 ± 6	7 ± 4	5 ± 5	4 ± 3

Primary astrocyte cultures were incubated for 5 h with 20 mM glucose without or with 1 mM pyruvate in the absence or the presence of 260 U catalase, 100 U SOD or 260 U catalase plus 100 U SOD before the extracellular concentrations of pyruvate and lactate as well as the extracellular LDH activity were determined. The data shown are means ± SD obtained from three experiments performed on independently prepared cultures

The initial cellular protein content was 157 ± 2 µg/well

No significant differences (ANOVA) compared to the values obtained for the control condition (None) were observed

### Substrate-Dependency of Extracellular Pyruvate and Lactate Accumulation in Astrocyte Cultures

**Fig. 4:**
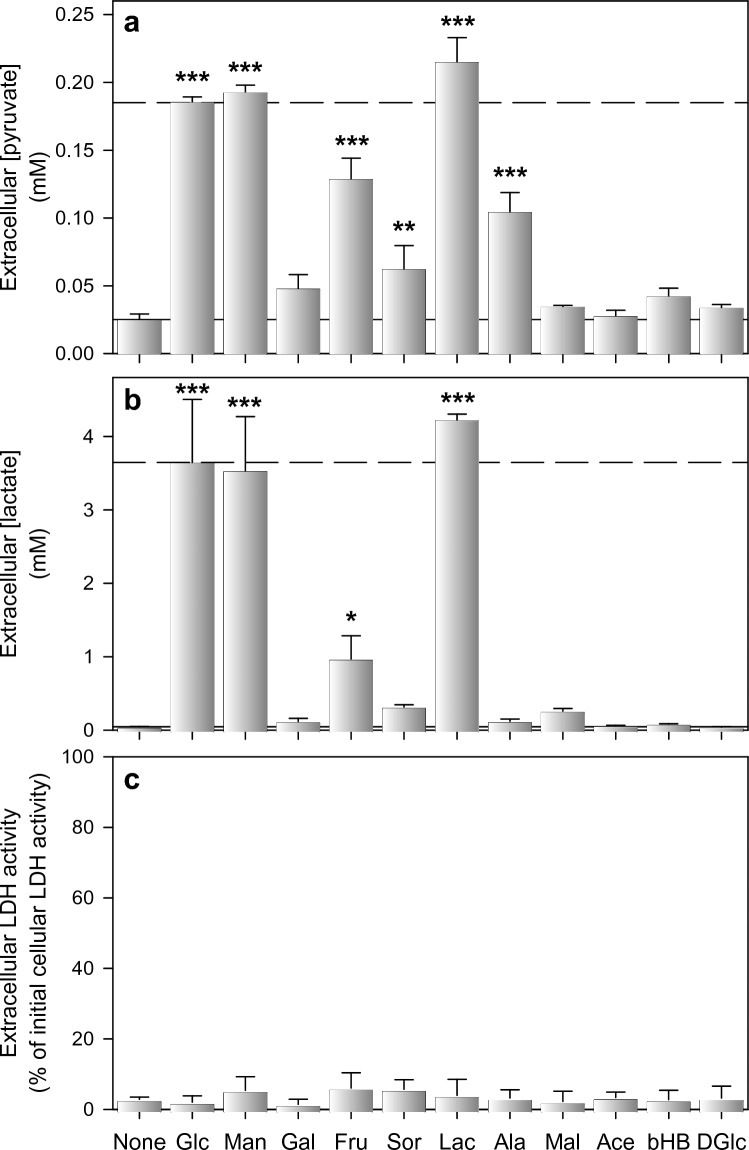
Extracellular accumulation of pyruvate and lactate after exposure of cultured astrocytes to different substrates. The cells were incubated for 5 h in glucose-free incubation buffer containing 5 mM of the indicated substrates before the extracellular concentrations of pyruvate (**a**) and lactate (**b**) as well as the extracellular LDH activity (**c**) were determined. The initial intracellular LDH activity was 170 ± 33 nmol/(min × well) and the initial protein content was 155 ± 19 µg/well. The data shown are means ± SD of values obtained in three experiments performed on independently prepared astrocyte cultures. The significance of differences (ANOVA) compared to the values obtained without any substrate (None) is indicated by *p < 0.05, **p < 0.01 and ***p < 0.001. Glc, glucose; Man, mannose; Gal, galactose; Fru, Fructose; Sor, sorbitol; Lac, lactate; Ala; alanine; Mal, Malate; Ace, acetate; bHB, β-hydroxybutyrate; DGlc, 2-deoxyglucose

Lactate and pyruvate are efficiently released from glucose-fed astrocytes (Figs. 1–3). To test for a potential release of pyruvate from astrocytes that had been exposed to other metabolic substrates than glucose, the cells were incubated for 5 h in 250 µL glucose-free buffer that had been supplemented with 5 mM of other hexoses or known mitochondrial substrates [18, 33, 60]. Compared to glucose-fed astrocytes, almost identical extracellular pyruvate concentrations were found for cells that had been exposed to mannose or lactate, but the cell established also in the presence of fructose, sorbitol or alanine extracellular pyruvate concentrations that were significantly higher than those determined for the substrate-free incubation (None) (Fig. 4a). Significantly increased extracellular lactate concentrations compared to the substrate-free condition were found for incubations with glucose, mannose, fructose and lactate (Fig. 4b). None of the conditions applied had any obvious toxic potential as indicated by the absence of any LDH release from the cells during the 5 h incubation (Fig. 4c).

**Fig. 5:**
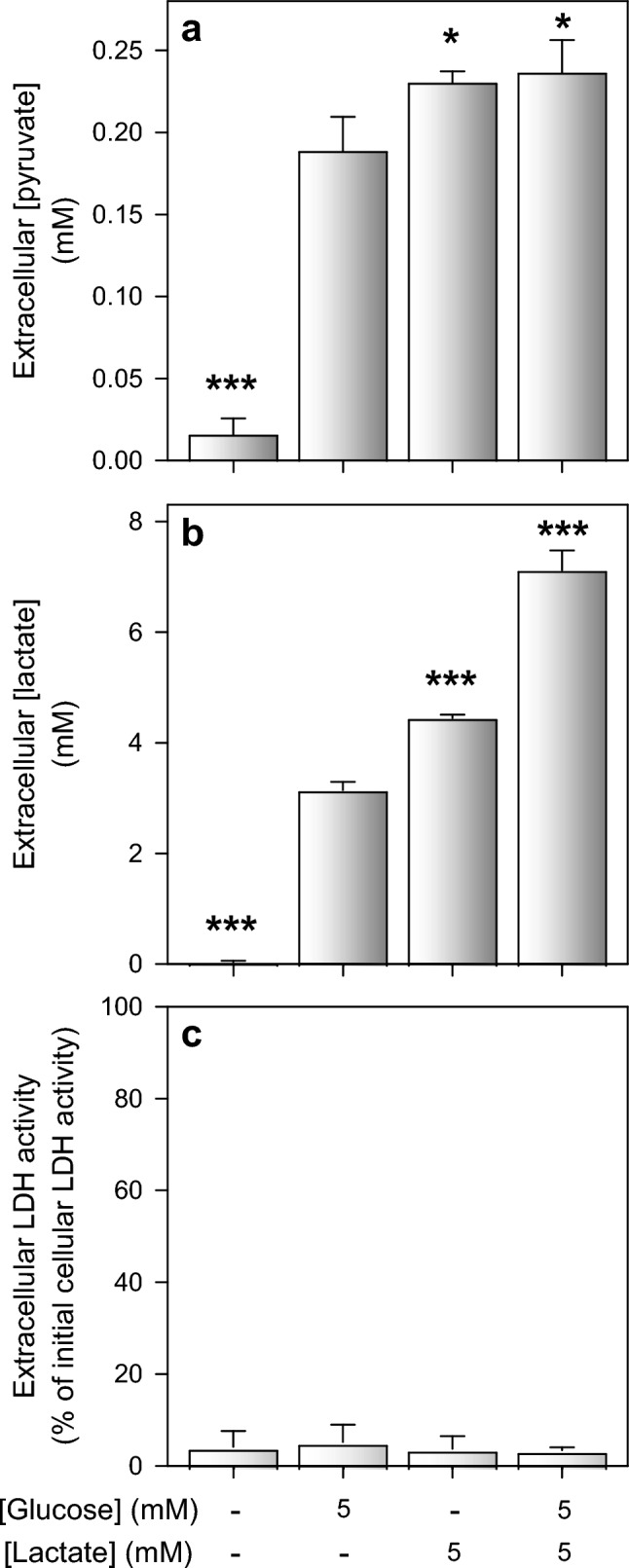
Extracellular pyruvate accumulation during incubation with lactate and/or glucose. Cultured primary rat astrocytes were incubated in the absence or the presence of 5 mM glucose and/or 5 mM lactate. After an incubation period of 5 h, the extracellular concentrations of pyruvate (**a**) and lactate (**b**) as well as the extracellular LDH activity (**c**) were determined. The initial cellular LDH activity of the astrocyte cultures was 130 ± 5 nmol/(min × well) and the initial protein content of the cultures was 156 ± 17 µg/well. The data shown are means ± SD of values that have been obtained in three experiments on independently prepared cultures. The significance of differences (ANOVA) compared to the values obtained for the glucose only condition is indicated by *p < 0.05 and ***p < 0.001

Astrocytes that had been incubated with either glucose or lactate release similar amounts of pyruvate (Fig. 5). To test whether pyruvate-formation and release by the presence of glucose and lactate may have a potential additive effect on the extracellular pyruvate accumulation, the cells were incubated without or with 5 mM of glucose and/or lactate. Although the extracellular lactate concentration determined after the incubation with lactate plus glucose were almost identical to the sum of lactate found for incubations with the individual substrates (Fig. 5b), similar concentrations of extracellular pyruvate of around 200 µM were found for the incubation with either glucose or lactate or with both substrates (Fig. 5a), demonstrating that extracellular pyruvate accumulation that is derived from the metabolism of glucose and lactate is not additive. None of the conditions applied had any obvious toxic potential as indicated by the absence of any LDH release from the cells during the 5 h incubation (Fig. 5c).

### Modulation of Extracellular Pyruvate Concentrations by Inhibitors of Pyruvate Transporters

**Fig. 6:**
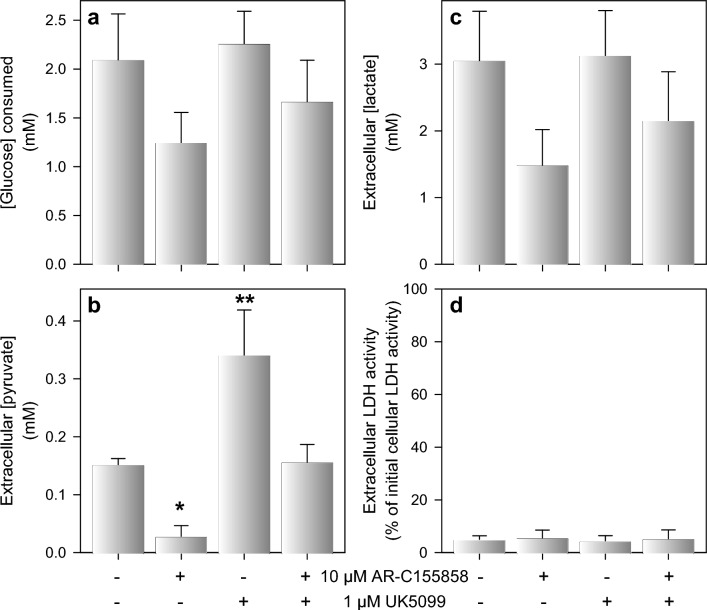
**Modulation of the extracellular pyruvate and lactate concentrations by inhibitors of monocarboxylate transporters.** Astrocyte cultures were incubated with 5 mM of glucose in the absence or presence of 10 µM of the MCT1 inhibitor AR-C155858 and/or 1 µM of the mitochondrial pyruvate carrier inhibitor UK5099. After 5 h of incubation, glucose consumption (**a**) and the concentrations of extracellular pyruvate (**b**) and lactate (**c**) were determined. In addition, extracellular LDH activity (**d**), as an indicator of a potential loss in cell viability, was measured. The initial intracellular LDH activity was 149 ± 40 nmol/(min × well) and the culture had an initial protein value of 134 ± 26 µg/well. The data represent means ± SD of data obtained in three experiments performed on independently prepared cultures. The significance of differences (ANOVA) compared to the values obtained for the control condition (no inhibitor) is indicated by *p < 0.05 and **p < 0.01

Pyruvate transport through the plasma membrane of astrocytes is mainly mediated by MCT1 [33, 37] while the mitochondrial MPC is involved in the uptake of cytosolic pyruvate into astrocytic mitochondria [18, 33, 61]. To test whether MCT1- and/or MPC-mediated transport may interfere with the extracellular pyruvate accumulation of glucose-fed astrocytes, the cells were incubated for 5 h in the absence or the presence of the MCT1 inhibitor AR-C155858 [62–64] and/or the MPC inhibitor UK5099 [24, 65, 66]. The presence of the MCT1 inhibitor lowered the cellular glucose consumption and lactate production by around 40%, while the MPC inhibitor did not affect these processes and was at best partially able to prevent the inhibitory potential of the AR-C155858 treatment (Fig. 6a, b). In contrast, the presence of the MCT1 inhibitor lowered significantly the extracellular pyruvate concentration by around 80%, while inhibition of MPC doubled the extracellular pyruvate concentration. The co-application of both inhibitors eliminated the strong effects observed for the individual inhibitors on the extracellular pyruvate concentration (Fig. 6c). None of the conditions applied had any obvious toxic potential as indicated by the absence of any LDH release from the cells during the incubation (Fig. 6d).

### Consequences of a Modulation of Mitochondrial Metabolism on the Extracellular Pyruvate Concentration of Astrocyte Cultures

**Fig. 7:**
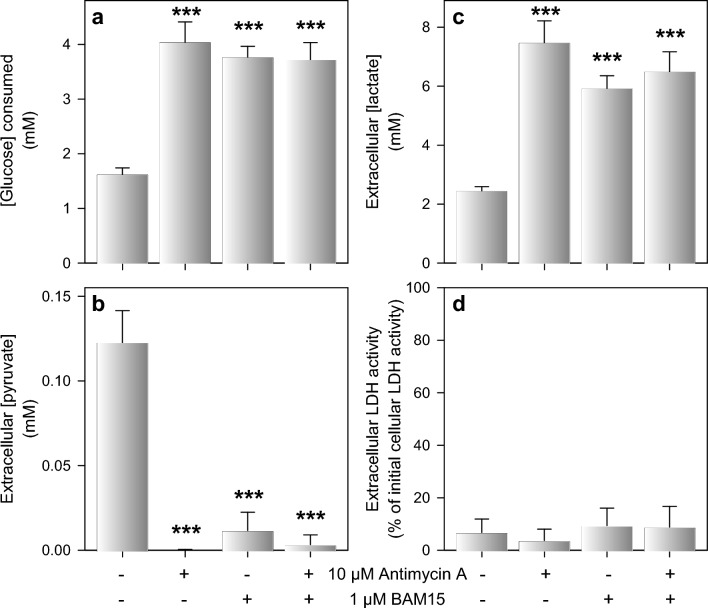
Alteration of extracellular pyruvate and lactate concentrations by application of the mitochondrial modulators antimycin A and/or BAM15. Astrocyte primary cultures were incubated with 5 mM glucose in the absence or the presence of 10 µM of the complex III inhibitor antimycin A and/or 1 µM of the uncoupler BAM15. After 5 h of incubation, glucose consumption (**a**), extracellular concentrations of pyruvate (**b**) and lactate (**c**) as well as the extracellular LDH activity (**d**) were determined. The cultures had an initial cellular LDH activity of 159 ± 31 nmol/(min × well) and an initial protein content of 148 ± 12 µg/well. The data shown represent means ± SD of data obtained in three experiments performed on individually prepared cultures. The significance of differences (ANOVA) compared to the values obtained for the control condition (no inhibitor or uncoupler) is indicated by ***p < 0.001

To investigate whether a modulation of mitochondrial metabolism may affect the extracellular pyruvate concentration in cultured astrocytes, the cells were incubated with 5 mM glucose in the presence of the complex III inhibitor antimycin A [67, 68] and/or the respiratory chain uncoupler BAM15 [69]. Exposure of cultured astrocytes for 5 h to those substances did not cause acute toxicity as indicated by the absence of any significant increase in extracellular LDH activity (Fig. 7d). Presence of antimycin A and BAM15 as well as the co-application of both compounds strongly increased glycolytic lactate production in cultured astrocytes as demonstrated by high values for glucose consumption (Fig. 7a) and lactate accumulation (Fig. 7c) that were more than doubled compared to the values for the control condition. In contrast, antimycin A and BAM15 almost completely diminished the extracellular pyruvate accumulation (Fig. 7b).

## Discussion

Pyruvate is an important metabolite that can be taken up [32, 33, 70] and released [34–36] by astrocytes. To investigate the processes that may affect astrocytic pyruvate release we have used astrocyte-rich primary cultures as a model system. During long-time incubations in DMEM culture medium, glucose-fed astrocytes released within the first day of incubation pyruvate to establish an extracellular concentration of around 300 µM that remained almost constant for days, at least if the cells had been exposed to a high concentration of glucose. This observation confirms literature data that report similar extracellular steady state concentrations in astrocyte-conditioned media during incubation of astrocytes for periods of up to 1 d [35] or 2 d [36]. However, the formation of an extracellular steady state pyruvate concentration appears not to be an exclusive feature of astrocytes, as it has also been reported for cultured neurons [35, 36] and for several cell lines of peripheral origin [53].

In contrast to the transient extracellular pyruvate accumulation, cultured astrocytes produced large amounts of lactate that accumulated almost proportional to time till the extracellular glucose had been consumed. As soon as the applied glucose had been metabolized, the cells started to consume the extracellular lactate that had been generated by glycolytic glucose metabolism, as previously reported [31], but also the extracellular pyruvate was consumed by the cells. Some decline in the extracellular steady state pyruvate concentration was also observed during extended incubation in DMEM supplemented with 10 mM glucose before the extracellular glucose had been completely metabolized. This partial decline in extracellular pyruvate concentration is consistent with the age-dependent tendency of cultured astrocytes to accumulate less extracellular pyruvate with increasing culture age (Fig. S1). Whether an increased mitochondrial activity or other reasons contribute to this age-dependent decline of extracellular pyruvate accumulation remains to be elucidated.

Pyruvate that was produced and released during incubation of cultured primary astrocytes in glucose-containing Hepes-buffered IB accumulated rapidly in the medium for around 5 h and established a transient extracellular steady state concentration of around 150–200 µM that was maintained for around 5 h. Reason for the discrepancy in the levels and the maintenance of extracellular pyruvate steady state concentrations observed for complex incubation media (DMEM) and the more simple HEPES-buffered IB appears to be mainly the use of the different buffer systems, as the extracellular pyruvate accumulation was also found accelerated in bicarbonate-buffered IB (Fig. S3). However, the more complex composition of the DMEM compared to IB affected also to some extent the extracellular pyruvate level as demonstrated by the doubling in extracellular pyruvate concentration in HEPES-buffered DMEM compared to HEPES-buffered IB (Fig. S3).

As excess of lactate has been shown to lower pyruvate consumption at least in glucose-deprived astrocytes [33], the accumulation of large extracellular concentrations of glucose-derived lactate should lower pyruvate uptake during extended incubation periods, thereby increasing extracellular pyruvate concentration. However, this appears not to be the case as the extracellular pyruvate concentration declined from a given transient steady state concentration in both DMEM- and IB-treated astrocytes after substantial amounts of the applied glucose had been metabolized. As lactate had accumulated under such conditions to millimolar extracellular concentrations, and as the cells are able to generate and export pyruvate from lactate as extracellular substrate, other factors than the available concentrations of the substrates lactate and glucose appear to be responsible for the observed decline in extracellular pyruvate concentration during extended incubation periods. For example, the absence of amino acids in IB, which could serve as anaplerotic substrates for citric acid cycle intermediates, may cause a delayed stimulated mitochondrial pyruvate consumption, thereby lowering cytosolic pyruvate concentration and subsequently stimulating consumption of extracellular pyruvate. At least for incubations in DMEM, a redistribution of mitochondria has been reported for the cells in astrocyte cultures after metabolic glucose-depletion that requires a metabolic shift from glycolytic glucose metabolism to oxidative lactate metabolism [31].

The delayed decline in detectable extracellular pyruvate levels over time could also be caused be the ability of extracellular pyruvate to chemically react with cell-derived hydrogen peroxide [52] which has been reported to protect neurons against toxicity induced by hydrogen peroxide [36, 43]. For peripheral cell lines, the detectable extracellular pyruvate level has been reported to be lowered to some extent by such a reaction [53]. However, although cultured astrocytes have frequently been reported to release hydrogen peroxide [55–57], the extracellular pyruvate accumulation and the detectable extracellular pyruvate concentration in cultured astrocytes were under the conditions used not affected by the presence of high activities of catalase and/or SOD. Thus, extracellular peroxide clearance by pyruvate can be excluded to contribute to the establishment of the transient steady state concentration of extracellular pyruvate in cultured astrocytes.

The initial constant velocity of pyruvate release from glucose-fed astrocytes suggests that pyruvate production by glycolysis and pyruvate consuming reactions establish for both incubation media almost constant intracellular pyruvate concentrations that define the velocity of pyruvate export. As pyruvate is transported through the plasma membrane of astrocytes mainly by the proton co-transporter MCT1 [33, 37], the proton gradient between the cytosol and the extracellular environment could affect pyruvate export and import. However, as the intracellular pH of cultured astrocytes rapidly adapts within minutes to the applied extracellular pH [71], it can be assumed that the intracellular pH remains rather constant during incubations of glucose-fed astrocytes under the conditions used and that the concentration gradient of pyruvate is the main driving force for its net transport over the astrocytic cell membrane.

We were unable to directly quantify cellular pyruvate contents for cultured astrocytes as cytosolic pyruvate concentrations are rather low in the micromolar range as demonstrated by genetically encoded sensors for cultured mouse astrocytes [24] or pyruvate-exposed HEK293 cells [70]. However, we at least calculated cytosolic pyruvate concentrations from the initial pyruvate release rates for glucose-fed astrocytes in HEPES-buffered IB (68 nmol/(h × mg)) and bicarbonate-buffered DMEM (108 nmol/(h × mg) (Fig. S3; means ± SD of data obtained in three experiments performed on independently prepared cultures). By using the Michaelis–Menten equation with the kinetic parameters previously determined for pyruvate transport in cultured astrocytes (Vmax = 7.5 nmol/(min × mg), K_M_ = 1 mM [32]), cytosolic pyruvate concentrations of 177 µM (IB) and 316 µM (DMEM) were calculated which fit quite well to the transient extracellular pyruvate steady state concentrations determined for glucose-fed cultured astrocytes in the respective media. The proposed adjustment of the extracellular steady state concentration of pyruvate to the intracellular pyruvate concentration would also explain why astrocytes consume excess of extracellular pyruvate to reach an appropriate extracellular pyruvate concentration. All these data support the view that the pyruvate concentration gradient is the main driving force for the extracellular accumulation of pyruvate and that net export of pyruvate is ceased after the extracellular concentration has reached the cytosolic pyruvate concentration.

Although both pyruvate and lactate are transported in astrocytes mainly by MCT1 [33, 37, 38, 72], the extracellular accumulation of pyruvate and lactate differ substantially. Extracellular pyruvate reached a transient stead state concentration in the micromolar range within hours while the extracellular lactate concentration continued to increase into the millimolar concentration range without reaching an extracellular steady state concentration. This observation, which confirms literature data [35], is likely to be the consequence of substantial differences in the cellular concentrations of both monocarboxylates as expected from the thermodynamic equilibrium of the LDH-catalyzed reaction [30]. Indeed, a specific cellular lactate content of 25.7 ± 2.1 nmol/mg (data from 4 experiments performed on independently prepared cultures) was determined for untreated cultured astrocytes and this value was not significantly altered by a 5 h incubation in IB with 10 mM glucose (data not shown). By using the specific cytosolic volume of 4.1 µL/mg protein [73], the cytosolic lactate concentration of untreated astrocytes was calculated to be around 6 mM. Thus, considering this high concentration of cytosolic lactate it is not surprising that lactate continues to accumulate in the extracellular medium of cultured astrocytes to the millimolar concentration range.

Assuming that the cytosolic pyruvate concentration is the main regulator of the extracellular pyruvate concentration in astrocyte cultures, at least for the initial phases of the incubations till a maximal extracellular pyruvate concentration has been reached, all treatments which modulate cellular pyruvate transport or metabolism should also affect the extracellular pyruvate concentration. Accordingly, in glucose-deprived astrocytes substantial concentrations of extracellular pyruvate were only found for incubations with either lactate or with other exogenous substrates that have previously been reported to be converted by astrocytes to lactate via pyruvate, including mannose, fructose, sorbitol and alanine [74–78]. In contrast, neither extracellular pyruvate nor lactate were found in glucose-deprived astrocytes that had been exposed to substrates such as acetate or beta-hydroxybutyrate which are unable to serve as precursors for pyruvate net synthesis but can be consumed for mitochondrial ATP synthesis in astrocytes [17, 18].

Pharmacological modulation of transporters and metabolic pathways that are known to modulate astrocytic pyruvate consumption [33] strongly affected the extracellular pyruvate concentration in glucose-fed astrocyte cultures. An increased extracellular pyruvate concentration was found for cultures that had been treated with UK5099, an inhibitor of mitochondrial pyruvate uptake [24, 33, 66], consistent with the importance of this transporter for mitochondrial pyruvate consumption [18, 33]. In contrast, extracellular pyruvate concentrations were severely lowered in glucose-fed astrocytes that had been treated with the uncoupler BAM15 or the respiratory chain inhibitor antimycin A. This can be explained for BAM15-treated astrocytes by a lowered cytosolic pyruvate concentration due to the accelerated mitochondrial pyruvate consumption reported for BAM15-treated astrocytes [33, 69]. In addition, the observed doubling of glycolytic lactate production by antimycin A, which confirms literature data [31, 68], and by BAM15 demonstrates for these conditions an increased cytosolic pyruvate consumption by LDH to regenerate the NAD^+^ needed to enable continuous glycolytic ATP regeneration. Extracellular pyruvate accumulation was also strongly inhibited by the MCT1 inhibitor AR-C155858, consistent with the function of MCT1 in pyruvate export and with the potential of this inhibitor to lower pyruvate consumption in astrocytes [33]. However, treatment with AR-C155858 lowered also glucose consumption and lactate release. Reason for this observation is most likely an acidification of the cellular pH by impairing proton-coupled export of glucose-derived lactate, consistent with the slower glycolytic glucose consumption in slightly acidified media (Fig. S4) [79, 80].

In conclusion, we have demonstrated that cultured astrocytes establish extracellular pyruvate concentrations that are likely to reflect their intracellular pyruvate concentrations which in turn depend on glycolytic pyruvate production, pyruvate reduction to lactate as well as on the mitochondrial pyruvate metabolism. A number of questions remains currently unanswered and should be addressed in future studies. A detailed analysis of the morphology and cellular composition of the astrocyte cultures after extended incubations in media with limited glucose concentrations should be done, for example by immunocytochemical characterization. Also, the molecular reasons underlying the observed decline in pyruvate export with age of the astrocyte cultures remain to be elucidated. Pyruvate release and extracellular pyruvate concentrations appear to be affected by multiple parameters, including the composition and pH of the incubation medium and the incubation time. It remains to be identified, which components in the incubation media applied are responsible for the different export rates for pyruvate and for the observed differences in the concentrations of pyruvate in astrocyte-conditioned media. Extracellular pyruvate concentrations are rapidly established, but they are transient and decline during longer incubations, although in many of the investigated conditions glucose is still available and large extracellular concentrations of glucose-derived lactate are present, which can also serve as exogenous substrate to generate pyruvate. The factors which are responsible for this decline in extracellular pyruvate are currently unknown and should be identified. Finally, the hypothesis that during the initial phase of incubation an equilibrium between cytosolic and extracellular pyruvate concentrations is established should be experimentally confirmed in a future study. For this, genetically encoded sensors [24] could be used and the detected cytosolic pyruvate concentrations could be directly correlated to the determined extracellular pyruvate concentrations for the various incubation conditions applied.

Cultured astrocytes establish a transient extracellular steady state concentration in the range of 150 to 300 µM which is quite similar to extracellular pyruvate concentrations (around 160 µM) that have been reported for brain tissue [46, 47] and the cerebrospinal fluid (CSF) (between 30 and 200 µM) [36, 81–83]. Due to their ability to release and consume pyruvate, it appears likely that astrocytes contribute to the reported extracellular pyruvate concentrations in brain and CSF. Pyruvate has been reported to by neuroprotective in neuropathological conditions such as glutamate-toxicity [41, 42], oxidative stress [36, 43] and ischemia [44] and several potential mechanisms for these neuroprotective functions have been discussed [45]. Pyruvate export has also been reported for cultured neurons, although the established extracellular pyruvate concentrations are lower than those found for astrocytes [35, 36]. Further in vivo studies are required to elucidate to which extent astrocytes contribute to the reported extracellular concentration of pyruvate in brain and CSF and whether a modulation of astrocytic pyruvate metabolism and release will have consequences for extracellular pyruvate levels in brain and/or on the neuroprotective potential of astrocytes.

### Supplementary Information

Below is the link to the electronic supplementary material.

Supplementary file1 (DOCX 303 KB)

## Data Availability

Enquiries about data availability should be directed to the authors.
