# Supplementary material for: Modulation of Pyruvate Export and Extracellular Pyruvate Concentration in Primary Astrocyte Cultures
Source: Neurochem Res. 2024 Feb 20;49(5):1331–46. doi: 10.1007/s11064-024-04120-0 (PMC10991036; doi:10.1007/s11064-024-04120-0)
Supplement: Supplementary file 1 — Supplementary file1 (DOCX 303 KB) [file 11064_2024_4120_MOESM1_ESM.docx]

**Modulation of pyruvate export and extracellular pyruvate concentration in primary astrocyte cultures**

**Nadine Denker and Ralf Dringen**

**Supporting Information**

**Figure S1**

**Figure S1: Effect of culture age on glucose consumption and the release of lactate and pyruvate from astrocytes.** Primary astrocyte cultures were incubated in an incubation medium containing 5 mM of glucose for 5 h. Afterwards, the respective initial protein contents (a), the initial cellular LDH activities (e). the glucose consumption in mM (b) as well as the extracellular concentrations of lactate (c) and pyruvate (d) were determined. Panels f, g and h give the specific values for the glucose consumption (f) and the extracellular accumulations of lactate (g) or pyruvate (h). The data obtained were used to calculate the ratios of lactate release to the glucose consumption (i), the ratio of the sum of pyruvate plus lactate release to glucose consumption (j), the ratio of glucose consumption to pyruvate release (k) and the ratio of lactate release to pyruvate release (l). The data are derived from a total of 36 experiments performed on 25 independently prepared cultures. Each data point represents the result of one individual experiment. Lines in the panels are derived from first order regression analyses of the data obtained and the respective correlation parameters are given in the individual panels.

**Figure S2**

**
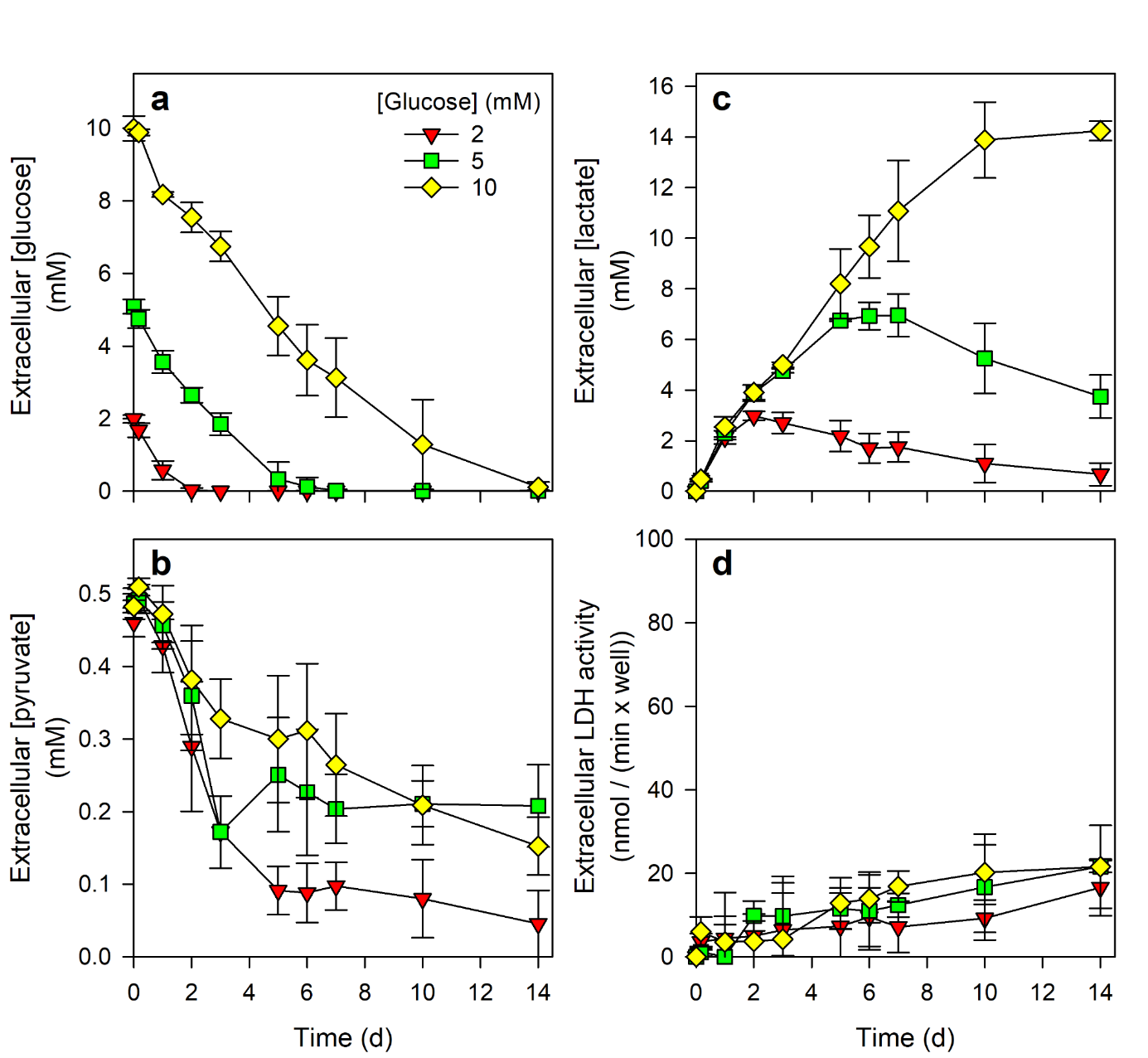
**

**Figure S2: Glucose consumption and extracellular concentrations of pyruvate and lactate in pyruvate-treated primary astrocyte cultures.** Astrocyte cultures were incubated with serum-free DMEM that contained pyruvate in an initial concentration of 0.5 mM and glucose in the initial concentrations indicated in panel a for up to 14 days. The extracellular concentrations of glucose (a), pyruvate (b) and lactate (c) as well as the extracellular LDH activity (d) were measured for the indicated time points. The initial cellular LDH activity of the cultures at the onset of the incubation (100%) was 106 ± 19 nmol/(min x well) and the initial protein content was 121 ± 55 µg/well. The data presented are means ± SD of data obtained in three individually performed experiments on independently prepared cultures.

**Figure S3**

**Figure S3: Glucose consumption and accumulation of pyruvate and lactate in different incubation buffers.** Primary rat astrocyte cultures were incubated with 10 mM glucose for up to 10 h in incubation buffer (145 mM NaCl, 5.4 mM KCl, 1.8 mM CaCl_2_, 1 mM MgCl_2_, 0.8 mM Na_2_HPO_4_) that had been buffered at 37°C to pH 7.4 with 20 mM HEPES/NaOH (IB-HEPES) or with 44.6 mM sodium bicarbonate/10% CO_2_ (containing 40 µM phenol red as pH indicator; IB-Bicarbonate). In addition, cultures were incubated in pyruvate-free DMEM that had been buffered either with 20 mM HEPES/NaOH (DMEM-HEPES) or with 44.6 mM sodium bicarbonate/10% CO_2_ (DMEM-Bicarbonate). The incubations in bicarbonate-buffered solutions were performed at 37°C in an incubator providing 10% CO_2_, while incubations in HEPES-buffered solutions were performed at 37°C in an incubator without CO_2_ supply. For the indicated incubation periods, the extracellular concentrations of glucose (a, e), lactate (b, f) and pyruvate (c, g) as well as the extracellular activity of LDH (d, h) were determined. The initial protein content of the cultures was 136 ± 7 µg/well and the initial cellular LDH activity was 209 ± 22 nmol/(min x well). The significance of difference (paired t-test) between values obtained for incubations in HEPES- and bicarbonate-buffered IB (e-h) is indicated by ^#^p<0.05 and ^##^p<0.01, that between bicarbonate-buffered DMEM (a-d) and bicarbonate-buffered IB (e-f) by ^+^p<0.05 and that between HEPES-buffered DMEM (a-d) and HEPES-buffered IB (e-h) is indicated by ^*^p<0.05. No significant differences (p<0.05) were observed for the data obtained for incubations with HEPES- and bicarbonate-buffered DMEM (a-d).

**Figure S4**

**Figure S4: Glucose consumption and extracellular pyruvate and lactate accumulation in incubation buffers of different pH values.** Astrocytes were incubated for up to 10 h in HEPES-buffered incubation buffers (containing 10 mM glucose) that had been adjusted to the pH values 7.0, 7.4 or 7.8. After the indicated time points, the extracellular concentrations of glucose (a), pyruvate (b) and lactate (c) as well as the extracellular LDH activity (d) were determined. The initial cellular LDH activity of the cultures was 224 ± 19 nmol/(min x well) and the initial protein content was 158 ± 19 µg/well. The significance of differences (ANOVA) compared to the values obtained for the control condition (pH 7.4) is indicated by ^*^p<0.05, ^**^p<0.01 and ***p<0.001.
